# In Vitro Antimicrobial Activity of Essential Oil Extracted from Leaves of *Leoheo domatiophorus* Chaowasku, D.T. Ngo and H.T. Le in Vietnam

**DOI:** 10.3390/plants9040453

**Published:** 2020-04-03

**Authors:** Nhan Trong Le, Duc Viet Ho, Tuan Quoc Doan, Anh Tuan Le, Ain Raal, Donatella Usai, Silvia Madeddu, Mauro Marchetti, Marianna Usai, Paola Rappelli, Nicia Diaz, Stefania Zanetti, Hoai Thi Nguyen, Piero Cappuccinelli, Matthew Gavino Donadu

**Affiliations:** 1 Faculty of Pharmacy, Hue University of Medicine and Pharmacy, Hue University, Hue 49000, Viet Nam; letrongnhan41@gmail.com (N.T.L.); hovietduc661985@gmail.com (D.V.H.); quoctuanqt91@gmail.com (T.Q.D.); 2 Mientrung Institute for Scientific Research, VAST, Hue 49000, Viet Nam; tasa207@gmail.com; 3 Institute of Pharmacy, Faculty of Medicine, University of Tartu, 50900 Tartu, Estonia; ain.raal@ut.ee; 4 Department of Biomedical Sciences, University of Sassari, 07100 Sassari, Italy; dusai@uniss.it (D.U.); rappelli@uniss.it (P.R.); ndiaz@uniss.it (N.D.); zanettis@uniss.it (S.Z.); mdonadu@uniss.it (M.G.D.); 5 CNR, Institute of Biomolecular Chemistry, Li Punti, 07100 Sassari, Italy; mauro.marchetti@ss.icb.cnr.it; 6 Department of Chemistry and Pharmacy, University of Sassari, 07100 Sassari, Italy; dsfusai@uniss.it; 7 Department of Biomedical Science, University of Cagliari, 09042 Monserrato CA, Italy

**Keywords:** antimicrobial activity, new anti-infectious agents, essential oil, phytochemicals

## Abstract

The present study aimed to determine the antimicrobial activity and chemical composition of leaves-extracted essential oil of *Leoheo domatiophorus* Chaowasku, D.T. Ngo and H.T. Le (*L. domatiophorus)*, including antibacterial, antimycotic, antitrichomonas and antiviral effects. The essential oil was obtained using hydrodistillation, with an average yield of 0.34 ± 0.01% (*v/w*, dry leaves). There were 52 constituents as identified by GC/MS with available authentic standards, representing 96.74% of the entire leaves oil. The essential oil was comprised of three main components, namely viridiflorene (16.47%), (-)-δ-cadinene (15.58%) and γ-muurolene (8.00%). The oil showed good antimicrobial activities against several species: Gram-positive strains: *Staphylococcus aureus* (two strains) and *Enterococcus faecalis,* with Minimum Inhibitory Concentration (MIC) and Minimum Lethal Concentration (MLC) values from 0.25 to 1% (*v/v*); Gram-negative strains such as *Escherichia coli* (two strains), *Pseudomonas aeruginosa* (two strains) and *Klebsiella pneumoniae*, with MIC and MLC values between 2% and 8% (*v/v*); and finally *Candida* species, having MIC and MLC between 0.12 and 4% (*v/v*).Antitrichomonas activity of the oil was also undertaken, showing IC_50_, IC_90_ and MLC values of 0.008%, 0.016% and 0.03% (*v/v*), respectively, after 48h of incubation. The essential oil resultedin being completely ineffective against tested viruses, ssRNA+ (HIV-1, YFV, BVDV, Sb-1, CV-B4), ssRNA- (hRSVA2, VSV), dsRNA (Reo-1), and dsDNA (HSV-1, VV) viruses with EC_50_ values over 100 µg/mL. This is the first, yet comprehensive, scientific report about the chemical composition and pharmacological properties of the essential oil in *L. domatiophorus*.

## 1. Introduction 

Medicinal plants have received a great deal of scientific attention over the past decades due to their low toxicity, cost-effectiveness, and promising pharmacological properties [1]. A wide range of efforts have been made on researching the great potentials of plant-extracted phytochemicals as well as their influences on human health [1,2]. In this regard, the extractions of essential oils from plants have brought about numerous medicinal values attributed to their useful biological and pharmacological activities [1,3].

Essential oils are aromatic oily liquid compounds extracted from different parts of the plants [4]. They are a blending of various single compounds [5]. More than 3000 essential oils have been documented, among them 300 found to be used in many applications, such as pharmacy, food industry, cosmetics and perfumes [6]. Some of those have been proved to treat certain organ and systemic disorders [3]. Essential oils exhibited powerful antioxidant, antibacterial, antifungal, antiviral and anti-inflammatory properties [7,8]. Certain essential oils have been used for curing tumors [9]. Thereby, recent studies have encouraged the use of plant-based extracts, such as essential oils, for the treatment of infectious diseases to overcome the pressing antibiotic resistance, while reducing many side effects of chemically synthetic molecules [10].

Annonaceae family, commonly known as the custard apple family or soursop family, includes trees, shrubs deciduous or evergreen, with aromatic bark, leaves, flowers, and rarely lianas [11]. The family comprises 2106 accepted species and more than 130 genera [11]. Biba et al. [12] reported that the Annonaceae was one of the families with various species whose chemical and pharmacological activities were known the least about. The seed oils of this family were widely documented as a great source of edible oils, and used for soap production [12]. The flowers of some Annonaceae species were employed in perfume industry and folk medicine [12]. A large number of chemical constituents, such as alkaloids, acetogenins, flavonoids and essential oils, were extracted from many other parts of Annonaceae species [12].

Many species among the Annonaceae are odorous as a result of the presence of essential oils, mainly composed of mono and sesquiterpene compounds [13]. Fournier et al. [14] demonstrated that more than 150 studies were dedicated to the oils extracted from 50 species belonging to *Annona* and *Xylopia* genera. Most constituents identified were commonly recognized, such as α-pinene, β-pinene, limonene, p-cymene, β-caryophyllene, and caryophyllene oxide [14]. Other compounds were less common and more specific to some Annonaceae species [14]. Some work focused on studying the essential oils of Annnonaceae growing wild in Vietnam [15,16,17].

Among the species of the Annonaceae family, which is widely distributed in Vietnam, we attempted to investigate *Leoheo domatiophorus* Chaowasku, D.T. Ngo and H.T. Le. *L.domatiophorus* is a medium-sized to large trees shrub species and distributed in rainforests of central Vietnam [18]. *L. domatiophorus* is the only one in genus *Leoheo*, and documented as one of the endangered species in the Red List of IUCN [18]. Phylogenetic analyses strongly support the belonging of the *L. domatiophorus* to *Monocarpieae*, sister to genus *Monocarpia*. However, the absence of intramarginal leaf veins, axillary inflorescences in the *L. domatiophorus* warrants its recognition as a distinct genus of *Monocarpieae* [18]. To the best of our knowledge, this is the first study on the investigation of chemical composition and bioactivities of compounds extracted from *L. domatiophorus*.

Herein, we aimed to study the chemical composition of the essential oil extracted from leaves of *L. domatiophorus* collected in ThuaThien Hue Province, Vietnam, and at the same time, to assess their potential antimicrobial, antitrichomonas and antiviral activities.

## 2. Materials and Methods

### 2.1. Plant Material

*L. domatiophorus* were collected from Nam Dong District, Thua Thien Hue Province, Vietnam in May 2019 (16°07’11.2” N; 107°36’58.3” E) and were identified by Dr. Nguyen Tien Chinh, Vietnam National Museum of Nature. A voucher specimen (LD-04) was deposited at the Faculty of Pharmacy, Hue University of Medicine and Pharmacy, Vietnam.

### 2.2. Extraction of the Essential Oil

The leaves of *L. domatiophorus* (5 kg) were shredded and the essential oil was hydrodistilled for 3.5 h at ambient pressure using a Clevenger-type apparatus [19]. The extraction yields (based on three replications) were calculated as percentages on dry material. The oil was dried on Na_2_SO_4_ and stored in sealed vials, at 4 °C, ready for the chemical analysis.

### 2.3. Analysis of the Essential Oil

Three repeats of sample were examined by the using of a *Hewlett-Packard Model 5890A* GC furnished with a flame-ionization detector and fitted with a 60 m × 0.25 mm, thickness 0.25 μm *AT-5* fused SiO_2_ capillary column. The injector and detector temperatures were the same (280 °C). The column temperature was programmed from 50 to 135 °C at 5 °C/min (1 min), 5 °C/min to 225 °C (5 min), 5 °C/min to 260 °C, held for 10 min. The oil (0.1 μL each) was analyzed without dilution (using 2,6-dimethylphenol as an internal standard) and injected by a split/splitless automatic injector *HP 7673*, using He as a carrier gas. The percentage of each compound was referred to absolute weight using internal standard and response factors. The detector response factors (*RF*s) that were determined for key constituents relative to 2,6-dimethylphenol and given to other components based on the correspondence of functional groups and/or structures. 

MS analyses were carried out using an *Agilent Technologies model 7820A* associated with a MS detector *5977E MSD* (*Agilent*), at the same conditions used for GC analyses. The column was linked to the ion source of the mass spectrometer. Mass units were monitored from 10 to 900 at 70 eV. 

The retention indices (RI) of single compound were determined by co-injection with a homologous series of *n*-alkanes (C_9_–C_22_) under the same conditions to (the retention indexes was calculate with the generalized equation by Van del Dool and Kartz [20]).

Data were processed for ANOVA by means of the software MSTAT-C and mean separation was performed by application of the LSD test at *p* ≤ 0.05 level of significance.

### 2.4. Antimicrobial Activity

Herein, there were 12 bacterial strains being selected, including 5 Gram-negative strains, namely *Escherichia coli* ATCC 35218, *Escherichia coli* clinical, *Pseudomonas aeruginosa* ATCC 27853, *Pseudomonas aeruginosa* clinical and *Klebsiella pneumoniae* clinical;3 Gram-positive bacteria such as *Staphylococcus aureus* ATCC 43300, *Staphylococcus aureus* clinical and *Enterococcus faecalis* clinical; *Candida* spp. strains: *Candida albicans* 556 RM, *Candida glabrata* clinical, *Candida tropicalis* 1011 RM and *Candida parapsilosis* RM. Cultures were performed in appropriate media at 4 °C. The cells were cultivated at 37 °C on agar plates, for 18 h prior to experiments.

### 2.5. Determination of Minimum Inhibitory Concentration (MIC) and Minimum Lethal Concentration (MLC)

In order to establish the MIC and MLC of bacteria and *Candida* species, the broth dilution method was employed, as reported by the Clinical and Laboratory Standard Institute [21]. The inoculum was prepared by diluting colonies in salt solution at a concentration of 0.5 McFarland, then confirmed at a wavelength of 530 nm by a spectrophotometric. The sensitivity test was implemented in LB broth and RPMI-1640 medium using 96-well plates. The oil solutions were diluted to a range of concentrations from 16% (*v/v*) to 5×10^−4^% (*v/v*). After shaking, 100 μL of each oil dilution and 100 μL of bacterial/yeast suspension at a concentration of 10^6^ CFU/mL were added to each well, then incubated at 37 °C for 24 to 48 h. MIC values were determined by the lowest concentration of the essential oil in which bacterial growth is visibly inhibited after overnight incubation. In order to determine the MLC value, 10 μL were seeded on Mueller Hinton agar and Sabouraud Dextrose agar and the plates were incubated for 24 to 48 h at 37 °C. Minimal lethal concentration (MLC) was considered as the lowest concentration that reduces the viability of the initial microbial inoculum, by ≥99.9%. Each experiment was performed in duplicate and repeated three times.

### 2.6. Cultivation of Trichomonas Vaginalis

*Trichomonas vaginalis* were axenically cultured in vitro by daily passages in Diamond’s TYM medium, and modified by adding 20% FBS, 300 IU/mL penicillin G, 300 μg/mL streptomycin at 37 °C in a 5% carbon dioxide atmosphere. The medium was well-observed and renewed on a daily basis to remove any miscellaneous matter [22]. Viable trophozoites were counted in a hemocytometer. *T. vaginalis* was harvested at the mid logarithmic phase with more than 95% viable cells, by centrifugation at 500 rpm for 10 min. A standard inoculum of 2 × 10^5^ cells/mL was prepared [23].

### 2.7. Determination of Minimal Lethal Concentration (MLC), 50% Inhibitory Concentration (IC_50_) and 90% Inhibitory Concentration (IC_90_)

*L. domatiophorus* essential oil was diluted in Diamond’s TYM medium from 2% to 0.002% (*v/v*). 100 μL of microbial culture was added to 100 µL at each concentration of different samples in 96-well plates. Sterile distilled water was used as a growth control. The culture plate was placed in a regular 37 °C incubator and examined after 1, 4, 24 and 48 h. Viable *T. vaginalis* cells were identified and counted upon microscopy, based on the morphology and motility. The MLC was defined as the lowest essential oil concentration in which no motile organism was observed. The IC_50_ and IC_90_ values were considered as the oil concentration, in which 50% and ≥90% *T. vaginalis* cells were killed. A positive growth control, consisting of organisms in broth, and a negative sterility control consisting of uninoculated broth, were included for each assay. Each assay was repeated independently at least twice [23].

### 2.8. Cells and Viruses

CD4+ human T-cells containing an integrated HTLV-1 genome (MT-4); Madin Darby Bovine Kidney (MDBK) (ATCC CCL 22 (NBL-1) Bos Taurus); Baby Hamster Kidney (BHK-21) (ATCC CCL 10 (C-13) Mesocricetus auratus); Monkey kidney (Vero-76) (ATCC CRL 1587 Cercopithecus aethiops) were used for the cytotoxic and antiviral studies.

The following viruses were purchased from the American Type Culture Collection (ATCC), with the exception of Human Immunodeficiency Virus type-1 (HIV-1) and yellow fever virus (YFV):IIIB laboratory strain of HIV-1, was obtained from the supernatant of the persistently infected H9/IIIB cells (NIH 1983); yellow fever virus (YFV) (strain 17-D vaccine (Stamaril Pasteur J07B01)); bovine viral diarrhoea virus (BVDV) (strain NADL (ATCC VR-534)); coxsackie type B4 (CV-B4) (strain J.V.B. (ATCC VR-184)); human enterovirus C (poliovirus type-1 (Sb-1) (Sabin strain Chat (ATCC VR-1562)); vesicular stomatitis virus (VSV) (lab strain Indiana (ATCC VR 1540)); human respiratory syncytial virus (hRSV) (strain A2 (ATCC VR-1540)); reovirus type-1 (Reo-1) (simian virus 12, strain 3651 (ATCC VR- 214)), vaccinia virus (VV) (vaccine strain Elstree-Lister (ATCC VR-1549)); human herpes 1 (HSV-1) (strain KOS (ATCC VR-1493)).

Cell cultures were checked periodically for the absence of mycoplasma contamination with MycoTect Kit (Gibco). The virus stocks were maintained in our laboratory and propagated in appropriate cell lines and aliquots were stored at −80 °C until use.

### 2.9. Cytotoxicity Assays

Exponentially growing MT-4 cells were seeded in 96-well plates, at a density of 4 × 10^5^ cells/mL in RPMI-1640 medium, 100 units/mL penicillin G and 100 μg/mL streptomycin and supplemented with 10% fetal bovine serum (FBS). BHK-21 cells were seeded at 6 × 10^5^ cells/mL in 96-well plates, in Minimum Essential Medium with Earle’s salts (MEM-E), L-glutamine, 1mM sodium pyruvate and 25 mg/L kanamycin, supplemented with 10% fetal bovine serum (FBS). MDBK cells were seeded at 1 × 10^6^ cells/mL in 96-well plates, in Minimum Essential Medium with Earle’s salts (MEM-E), L-glutamine, 1 mM sodium pyruvate and 25mg/L kanamycin, supplemented with 10% horse serum. Vero-76 cells were seeded at an initial density of 5 × 10^5^ cells/mL in 96-well plates, in Dulbecco’s Modified Eagle Medium (D-MEM) with L-glutamine and 25 mg/L kanamycin, supplemented with 10% FBS. Cell cultures were then incubated at 37 °C with an atmosphere of 5% CO_2_, in the absence or presence of serial dilutions of test essential oil. The medium employed for the cytotoxic assay, as well as for the antiviral assay, contained 1% of the appropriate serum. Cell viability was verified at 37 °C by the 3-(4,5-dimethylthiazol-1-yl)-2,5-diphenyltetrazolium bromide (MTT) method after 72 h for BHK-21, MDBK and Vero-76 or 96 h for MT-4 [24]. The cytotoxic activity of leaves’ essential oil of *L. domatiophorus* was evaluated in parallel with its antiviral activity, through the viability of mock-infected, treated cells, as determined by the MTT method.

### 2.10. Antiviral Assays

Essential oil’s activity against HIV-1 was based on the inhibition of virus-induced cytopathogenicity in exponentially growing MT-4 cell acutely infected with a multiplicity of infection (m.o.i.) of 0.01. Briefly, 50 μL of RPMI-1640 containing 1 × 10^4^ MT-4 cells were added to each well of flat-bottom microtitre trays, containing 50 μL of RPMI-1640, with or without serial dilutions of the essential oil. Then, 20 μL of a HIV-1 suspension containing 100 CCID_50_ was added.

Essential oil’s activity against YFV, and Reo-1 was based on the inhibition of virus-induced cytopathogenicity in BHK-21 cells, acutely infected at an m.o.i. of 0.01. Essential oil’s activity against BVDV was based on inhibition of virus-induced cytopathogenicity in MDBK cells acutely infected at an m.o.i. of 0.01. Briefly, BHK and MDBK cells were seeded in 96-well plates, at a density of 5 × 10^4^ and 3 × 10^4^ cells/well, respectively, and were allowed to form confluent monolayers by incubating overnight in growth medium at 37 °C in a humidified CO_2_ (5%) atmosphere. Cell monolayers were then infected with 50 μL of a proper virus dilution in MEM-E, then 50 μL of medium, with or without serial dilutions of the essential oil, were added.

After a 3, or 4 -day incubation at 37 °C, cell viability was measured by the MTT method [24]. Compound’s activity against CV-B4, Sb-1, VV, VSV, hRSV A2 and HSV-1 was determined by plaque reduction assays in infected cell monolayers, as described previously [25]. Briefly, the monolayer of Vero-76 cells was grown overnight on a 24-well plate. The cells were then infected for 2 h with 250 μL of proper virus dilutions, to give 50-100 PFU/well. After the incubation period, the unadsorbed virus was removed, 500 μL of D-MEM containing 0.75% methyl-cellulose, with serial dilutions of test products, were added. The overlayed medium was also added to not treat wells as non-infection controls. Cultures were incubated at 37 °C for a period of 2 (Sb-1 and VSV), 3 days (CV-B4, hRSV A2, VV, and HSV-1), and then fixed with PBS containing 50% ethanol and 0.8% crystal violet, washed and air-dried. The number of plaques in the control (no inhibitor) and experimental wells were then counted.

### 2.11. Linear Regression Analysis

The extent of cell growth/viability and viral multiplication, at each drug concentration tested, were expressed as percentages of untreated controls. Concentrations resulting in 50% inhibition (CC_50_ or EC_50_) were determined by a linear regression analysis using data from three independent experiment performed in duplicate.

## 3. Results and Discussion

### 3.1. Extraction Yield and Chemical Composition of Essential Oil

In *L. domatiophorus* leaves, the average yield of hydrodistilled essential oil was of 0.34 ± 0.01%, calculated on a dry weigh of three samples. The obtained essential oil was a pale, yellow liquid with odor and lighter than water. The GC/MS analysis indicated that the leaves’ essential oil contained 52 constituents representing 96.74% of the total oil content (Table 1). The main classes of compounds in this oil were sesquiterpene hydrocarbons (74.04%), oxygenated sesquiterpenes (22.01%). The constituents accounted for higher amounts in the leaves oil of *L. domatiophorus* were viridiflorene (16.47%), (-)-δ-cadinene (15.58%) and γ-muurolene (8.00%). The other components found at lower concentration were α-muurolene (5.45%), γ-cadinene (5.18%), (+)-aromadendrene (3.82%), α-cadinol (3.59%) and globulol (3.10%). In addition, the oil also contained one aldehyde, nonanal (0.02%) and one alcohol, *cis*-α-ambrinol (0.51%), having a non-terpenic structure. To the best of our knowledge, this is the first scientific report about chemical composition of the essential oil from *L. domatiophorus_._*

### 3.2. Antimicrobial Activities

The antimicrobial activities of essential oil in *L. domatiophorus* were displayed in Table 2. The results exhibited potential antibacterial activities of the essential oil against i) Gram-positive bacterium: *S. aureus* (two strains) with MIC of 0.25% (*v/v*) and MLC of 0.5% (*v/v*), and *E. faecalis* with MIC and MLC were both 1% (*v/v*); Gram-negative bacteria such as *E. coli* (two strains) with MIC and MLC from 2 to 8% (*v/v*), *P. aeruginosa* (two strains) having MIC and MLC between 2 and 4% (*v/v*), and *K. pneumoniae* with MIC and MLC both equivalent to 4% (*v/v*); *Candida species* showed MIC and MLC as follows: *C. albicans,* with MIC and MLC were both 4% (*v/v*), *C. glabrata,* with MIC and MLC were both 2% (*v/v*), *C. tropicalis*, with MIC and MLC of 1% and *C. parapsilosis,* with MIC and MLC both equivalent to 0.12% (*v/v*).

### 3.3. Antitrichomonas Activity

As shown in Table 3, the leaves essential oil of *L. domatiophorus* showed potential in vitro antitrichomonas activity. Essential oil of *L. domatiophorus* indicated a remarkable antitrichomonas activity against *T. vaginalis*, and the values IC_50_, IC_90_, MLC were time-dependent at 1, 4, 24 and 48 h. The obtained results suggested the efficacy of the essential oil after 1 h of incubation, and trichomonas was strongly inhibited after 24h. In particular, the effect of the agent increased, the values IC_50_, IC_90_ and MLC of *L. domatiophorus* essential oil from leaves were 0.008, 0.016, and 0.03% (*v/v*), respectively, after 48 h of incubation. 

### 3.4. Antiviral Activity

Here, we explored the antiviral properties of leaves’ essential oil of *L. domatiophorus* against a broad spectrum of RNA and DNA viruses belonging to different families, including several important human pathogens. Among ssRNA+ viruses, were: human immunodeficiency virus type-1 [HIV-1] (Retroviridae); two Flaviviridae, bovine viral diarrhoea virus [BVDV] and yellow fever virus [YFV]; two Picornaviridae, human enterovirus B [coxsackie virus B4, CV-B4] and human enterovirus C [polio virus type-1, Sb-1]. Among ssRNA- viruses, were: human respiratory syncytial virus [hRSVA2] (Pneumoviridae) and vesicular stomatitis virus [VSV] (Rhabdoviridae). Among dsRNA viruses, we tested reovirus type-1 [Reo-1] (Reoviridae). Finally, representatives of two DNA virus families were also included: vaccinia virus [VV] (Poxviridae) and human herpesvirus 1 [herpes simplex type-1, HSV-1] (Herpesviridae). In order to be able to establish whether test *L. domatiophorus* oil were endowed with selective antiviral activity, their cytotoxicity was evaluated in parallel assays with uninfected cell lines. In vitro cytotoxicity was measured based on cell proliferation and viability. The CC_50_ (drug concentration inhibiting cell growth by 50% referred to untreated control) was > 100 µg/mL and no cell toxic effect was observed (Table 4). However, results obtained from our screening pointed out *L. domatiophorus* essential oil was completely ineffective against the tested viruses with EC_50_ values over 100 µg mL^−1^.

The Annonaceae family was documented by Jussieu in 1789 [13]. Their essential oils predominantly constituted monoterpenes, sesquiterpenes, and alkaloids, especially isoquinoline alkaloids [13]. Several compositions of essential oils have been commonly reported, for example α-pinene, β-pinene, limonene, p-cymene, β-caryophyllene and caryophyllene oxide [14]. Nonetheless, the components of essential oils can vary upon specific species and their distributions in distinct geographic regions [35,36]. In the present work, the main components of *L. domatiophorus* essential oil was reported, such as (-)-δ-cadinene, γ-muurolene and α-cadinol. These compounds were also found with high concentration in essential oils of some species belonging to Annonaceae family, namely *Anaxagoma dolichocarpa* [14], *Annona salzmannii* [37], *Annona muricata* [38], *Xylopia pynaertii* [39] and *Cananga odorata* [40]. Additionally, the main ingredients in essential oil of *Xylopia frutescens* were viridiflorene and δ-cadinene [41], while such of *Xylopia laevigata* were δ-cadinene and γ-muurolene [42]. Even though some of these volatile compounds can be present in essential oils of other families, the similarity of the main constituents in essential oils of species in the same family suggested that they have an important chemotaxonomic relevance [42].

Moreover, *L. domatiophorus* essential oil exhibited the strongest antimicrobial activity against *S. aureus* and *Candida* species, which was observed to be in line with previous studies on antimicrobial activity of Annonaceae species. Several work demonstrated that *A. salzmannii*, *A. senegalensis* and *X. aethiopica* essential oils could inhibit *S. aureus* [43,44,45], while such of *A. vepretorum* and *X. aethiopica* possessed the inhibiting capacity towards *Candida* species [44,46]. The biological activities of essential oils, mainly focused on the antibacterial, antifungal and antioxidant activities [1,3]. Only a few essential oils, especially some species of the Lamiaceae family, have been investigated against *Trichomonas* [47,48,49,50,51]. Notably, our work on the antitrichomonas activity of *L. domatiophorus* oil, for the first time, demonstrated an effective inhibition against *T. vaginalis*.

Until now, the efficacy of some essential oils has been explored against *S. aureus* and *C. parapsilosis*. A report of 105 clinical isolates showed *Melaleuca alternifolia* oil against *S. aureus* with MIC_90_ of 0.5% (*v/v*) [52]. A later study was conducted on 100 clinical isolates of methicillin-resistant *S. aureus* found the MIC_90_ of *M. alternifolia* oil at 0.32% (*v/v*) [52]. The antimicrobial activity against *S. aureus* of *Carum carvi*, *Pogostemon cablin* and *Pelargonium graveolens* essential oils were observed [53]. The MIC values of *C. carvi*, *P. cablin* and *P. graveolens* oils were 1.88 ± 1.03; 0.17 ± 0.08 and 0.54 ± 0.20% (*v/v*) respectively [53]. Herein however, it can be seen that *L. domatiophorus* oil displayed higher inhibition against *S.aureus* with MIC and MLC of 0.25% and 0.5% (*v/v*) respectively, compared to that of *M. alternifolia*, *C. carvi*, *P. cablin* and *P. graveolens* oils. According to Mondello *et al*, *M. alternifolia* oil inhibited *Candida* strains with MIC ranging from 0.03% to 0.25% [54]. On the contrary, *L. domatiophorus* essential oil displayed promising inhibition against *Candida* species, especially in *C. parapsilosis* with MIC and MLC values of 0.12% (*v/v*). As a result, *L. domatiophorus* oil is very likely to become an alternative therapeutic agent for infectious diseases, yet further microbiological tests and clinical trials should be assessed. 

Several types of plant-extracted compounds may display antimicrobial activities. They are synthesized to protect the plants from external pathogens [55]. Under particular biotic/abiotic stress conditions, their chemical constituents are released via a plethora of molecular interactions [56]. Each composition exhibits various mechanisms of actions against bacteria [57], thus exerting different effects on the ultimate antimicrobial properties of essential oils [58]. In other words, essential oils containing different chemical compositions are prone to disrupt bacteria in different pathways [55]. Additionally, hydrophobicity plays a crucial role in essential oils, contributing to an increased permeation through the cell membrane. This is likely to result in the spillage of ions and molecules, and consequently to cell death [59]. In general, Gram-positive bacteria are more sensitive to essential oils than Gram-negative bacteria because the cell wall of Gram-positive bacteria is less complex than Gram-negative ones [58,60]. In our study, the antimicrobial activities of the essential oil could potentially stem from their main components, such as (-)-δ-cadinene. Several studies have demonstrated that plants containing (-)-δ-cadinene as the main compound display good antibacterial activity [61,62,63,64]. In addition, the presence of many individual antimicrobial components in the essential oil is more likely to produce a synergy, and that amplifying the antimicrobial activities of the essential oil as a whole [35,65].

Multidrug resistance has become a huge challenge of healthcare worldwide, remaining one of the leading causes of human mortality over the past few years [66]. Bacteria, fungi and parasites have consecutively been developing numerous resistant mechanisms against current antibiotics, hampering the success of anti-infectious therapies, and thus leaving severe consequences on patients’ health [67,68,69,70]. In addition, the use of synthetic chemicals to control microorganisms is still limited due to their carcinogenic effects, acute toxicity and environmental hazards [55]. Hence, a demand for new antibiotics is urgently raised among scientific community to address multidrug resistance [58]. The therapeutic agents from herbal medicines have long emerged as a potential natural source for treating infectious diseases [23,71]. Herein, the antimicrobial, antitrichomonas and antiviral effects of essential oil from *L. domatiophorus* were first studied, showing strong activity against the studied strains, in particular *S. aureus, E. faecalis*, *Candida* species and *T. vaginalis.* There by, the essential oil of *L. domatiophorus* can be employed in the development of new anti-infectious agents, thanks to its strong bactericidal effects. 

## 4. Conclusions

The fresh leaves essential oil of *L. domatiophorus* after collecting from Thua Thien Hue Province, Vietnam was composed of 52 constituents in which viridiflorene (16.47%), (-)-δ-cadinene (15.58%) and γ-muurolene (8.00%) were three main components. *L. domatiophorus* essential oil displayed antimicrobial activities against two Gram-positive strains, *S. aureus* and *E. faecalis,* with MIC and MLC values from 0.25 to 1% (*v/v*); three Gram-negative bacteria, *E. coli*, *P. aeruginosa* and *K. pneumoniae*, with MIC and MLC values between 2 and 8% (*v/v*); and finally *Candida* species, having MIC and MLC between 0.12 and 4% (*v/v*). The oil also exhibited repellency against *T. vaginalis* with IC_50_, IC_90_ and MLC values of 0.016, 0.03 and 0.06% (*v/v*). It was ineffective against HIV-1, YFV, BVDV, Sb-1, CV-B4, hRSVA2, VSV, Reo-1, HSV-1, VV viruses. Further studies should be done to evaluate the safety and toxicity of *L. domatiophorus* essential oil in animals, before considering the development of new anti-infectious agents for use in clinical trials.

## Figures and Tables

**Table 1:** Chemical composition of the essential oil from the leaves of *L. domatiophorus.*

No.	RT	^a^KI	Components	^b^% ± SD	^c^IM	^d^References
1	22.17	1003	α-Phellandrene	0.02 ± 0.01	Std	
2	22.75	1019	α-Terpinene	0.01 ± 0.01	Std	
3	23.17	1025	m-Cymene	0.03 ± 0.01	Std	
4	23.43	1028	(+)-Limonene	0.02 ± 0.01	Std	
5	23.56	1032	β-Phellandrene	0.01 ± 0.01	Std	
6	24.26	1035	*cis*-β-Ocimene	0.01 ± 0.01	Std	
7	27.37	1103	Nonanal	0.02 ± 0.01	Std	
8	31.44	1179	Terpinen-4-ol	0.02 ± 0.01	Std	
9	32.14	1189	L-α-Terpineol	0.01 ± 0.01	Std	
10	36.24	1274	Phellandral	0.03 ± 0.01	MS	NIST
11	39.90	1376	α-Ylangene	0.24 ± 0.02	MS	NIST
12	39.95	1378	Isoledene	0.38 ± 0.02	MS	NIST
13	40.13	1379	α-Copaene	1.01 ± 0.03	Std	
14	40.55	1384	Z-β-Elemene	0.22 ± 0.03	Std	
15	41.45	1413	β-Maaliene	0.40 ± 0.02	MS-RI	[26]
16	41.72	1421	*E*-β-Caryophyllene	1.28 ± 0.04	Std	
17	41.95	1423	γ-Maaliene	0.26 ± 0.01	MS	NIST
18	42.23	1441	*cis*-α-Ambrinol	0.51 ± 0.02	MS	NIST
19	42.35	1440	(+)-Aromadendrene	3.82 ± 0.21	MS	NIST
20	42.47	1443	Cedrane	0.22 ± 0.01	MS	NIST
21	42.54	1451	*cis*-Muurola-3,5-diene	0.51 ± 0.01	MS	Adams
22	42.64	1451	α-Himachalene	0.21 ± 0.01	MS	NIST
23	42.79	1452	*trans*-Muurola-3,5-diene	0.27 ± 0.01	MS	Adams
24	42.88	1452	α-Humulene	0.44 ± 0.02	Std	
25	43.04	1463	Alloaromadendrene	0.74 ± 0.04	MS	NIST
26	43.40	1480	γ-Muurolene	8.00 ± 0.22	MS	[27]
27	43.52	1480	α-Muurolenediastereoisomer	2.92 ± 0.11	MS	NIST
28	43.66	1484	Germacrene D	1.77 ± 0.11	MS	Adams
29	43.78	1493	(-)-β-Cadinene	1.20 ± 0.09	MS	[28]
30	43.98	1496	Ledene (Viridiflorene)	16.47 ± 0.21	MS-RI	[29]
31	44.08	1501	α-Muurolene	5.45 ± 0.12	MS	[30]
32	44.24	1514	(+)-δ-Cadinene	1.31 ± 0.05	Std	
33	44.56	1514	γ-Cadinene	5.18 ± 0.11	Std	
34	44.67	1523	(-)-δ-Cadinene	15.58 ± 0.42	MS	Adams
35	44.78	1527	*trans*-Calamenene	1.60 ± 0.07	MS	NIST
36	44.83	1530	Epizonarene	0.90 ± 0.04	MS-RI	[31]
37	45.08	1533	Cada-1,4-diene	0.96 ± 0.07	MS-RI	[32]
38	45.20	1540	α-Muurolenediastereoisomer	1.82 ± 0.08	MS	NIST
39	45.36	1549	α-Calacorene	0.88 ± 0.04	MS	NIST
40	46.03	1568	Epiglobulol	1.00 ± 0.05	MS	NIST
41	46.28	1571	Palustrol	0.89 ± 0.04	MS-RI	[33]
42	46.43	1578	(+)-Spathulenol	0.97 ± 0.04	Std	
43	46.71	1583	Globulol	3.10 ± 0.12	MS	Adams
44	46.95	1591	Viridiflorol (Ledol)	2.03 ± 0.11	MS	NIST
45	47.21	1599	Rosifoliol	1.79 ± 0.08	MS	NIST
46	47.66	1608	Eudesmol<5epi-7-epi-α>	1.09 ± 0.07	MS	Adams
47	47.70	1616	Cubenol	2.20 ± 0.09	MS-RI	[34]
48	47.84	1619	(-)-Spathulenol	0.57 ± 0.04	Std	
49	48.04	1639	α-epi-Cadinol	1.65 ± 0.03	Std	
50	48.09	1640	α-epi-Muurolol	2.40 ± 0.12	Std	
51	48.13	1646	(-)-δ-Cadinol	0.73 ± 0.04	Std	
52	48.41	1654	α-Cadinol	3.59 ± 0.09	Std	
			**Total**	**96.74**		
			**Monoterpene hydrocarbons**	**0.1**		
			**Oxygenated monoterpenes**	**0.06**		
			**Sesquiterpene hydrocarbons**	**74.04**		
			**Oxygenated sesquiterpenes**	**22.01**		
			**Others**	**0.53**		

Data are the mean of three replicates ± SD.^a^Retention index (Kovalts) relative to *n*-alkanes (C_9_–C_22_). ^b^ Percentage of compounds. **^c^** Identification methods (IM): **MS** by comparison of the Mass spectrum with those of the computer mass libraries Adams, NIST 11 and by interpretation of the mass spectra fragmentations. **RI** by comparison of retention index with those reported in literature. **Std** by comparison of the retention time and mass spectrum of available authentic standards. **^d^** Papers take as reference to compare the relative RI.

**Table 2:** Antimicrobial activities (MIC and MLC) of essential oil from the leaves of *L. domatiophorus.*

Strains	^a^MIC (% *v/v*)	^b^MLC (% *v/v*)
**Gram-positive bacteria**		
*S. aureus* ATCC 43300	0.25	0.5
*S. aureus* clinical strain	0.25	0.5
*E. faecalis* clinical strain	1	1
**Gram-negative bacteria**		
*E. coli* ATCC 35218	4	8
*E. coli* clinical strain	2	4
*P. aeruginosa* ATCC 27853	2	4
*P. aeruginosa* clinical strain	4	4
*K. pneumoniae* clinical strain	4	4
**Yeast**		
*C. albicans*556 RM	4	4
*C. glabrata* clinical	2	2
*C. tropicalis*1011 RM	1	1
*C. parapsilosis* RM	0.12	0.12

^a^ MIC: Minimum Inhibitory Concentrations; ^b^ MLC: Minimum Lethal Concentrations.

**Table 3:** In vitro anti-*T.vaginalis* activity of essential oil from the leaves of *L. domatiophorus.*

Time	IC_50_ (% *v/v*)	IC_90_ (% *v/v*)	MLC (% *v/v*)
**1 h**	1	2	4
**4 h**	0.12	0.25	0.5
**24 h**	0.016	0.03	0.06
**48 h**	0.008	0.016	0.03

**IC_50_**: The concentration that causes 50% Trichomonas growth inhibition. **IC_90_:** The concentration that causes ≥ 90% Trichomonas growth inhibition. **MLC:** The concentration that causes the death of 100% Trichomonas.

**Table 4:** Cytotoxicity and antiviral activity of essential oil from *L. domatiophorus* against representatives of ssRNA^+^ (HIV-1,YFV, BVDV, Sb-1, CV- B4), ssRNA^−^ (hRSV A2, VSV), dsRNA (Reo-1), and dsDNA (HSV-1, VV) viruses.

Cell Lines and Virus	MT4	HIV-1_IIIB_	BHK-21	YFV	Reo-1	MDBK	BVDV	Vero-76	hRSVA2	VSV	HSV-1	VV	Sb-1	CV-B4
	CC_50_^a^	EC_50_^b^	CC_50_^c^	EC_50_^d^	EC_50_^d^	CC_50_^e^	EC_50_^f^	CC_50_^g^	EC_50_^h^					
Oil	>100	>100	>100	>100	>100	>100	>100	>100	>100	>100	>100	>100	>100	>100
*Reference Compds														
**RC1**	40	0.003 ± 0.0003	-	-	-	-	-	-	-	-	-	-	-	-
**RC2**	-	-	80	1.4 ± 0.2	-	>100	1.7 ± 0.3	-	-	-	-	-	-	-
**RC3**	-	-	-	-	-	-	-	>100	-	-	-	-	2	2 ±0.5
**RC4**	-	-	-	-	-	-	-	≥14	2 ± 0.2	-	-	-	-	-
**RC5**	-	-	-	-	-	-	-	>100	-	-	3.0 ± 0.1	-	-	-
**RC6**	-	-	-	-	-	-	-	19	-	-	-	1.7 ± 0.1	-	-
**RC7**	-	-	>100	-	17	-	-	-	-	-	-	-	-	-

Data represent mean values ŷ SD for three independent determinations. For values where SD is not shown, variation among duplicate samples was less than 15%. **Oil:** essential oil from the leaves of *L. domatiophorus;* **RC1:** Efavirenz; **RC2:** 2′-C-methylguanosine; **RC3**: Pleconaril; **RC4**: 6-aza-uridine; **RC5**: Acycloguanosine; **RC6**: Mycophenolic acid; **RC7**: 2′-C-methylcytidine; ^a^ compound concentration (µg mL^−1^) required to reduce the proliferation of mock-infected MT-4 cells by 50%, as determined by the MTT method. ^b^ Compound concentration (µg mL^−1^) required to achieve 50% protection of MT-4 cells from HIV-1 induced cytopathogenicity, as determined by the MTT method. ^c^ Compound concentration (µg mL^−1^) required to reduce the viability of mock-infected BHK-21 cells by 50%, as determined by the MTT method. ^d^ Compound concentration (µg mL^−1^) required to achieve 50% protection of BHK-21 cells from YFV or Reo-1 induced cytopathogenicity, as determined by the MTT method. ^e^ Compound concentration (µg mL^−1^) required to reduce the viability of mock-infected MDBK cells by 50%, as determined by the MTT method. ^f^ Compound concentration (µg mL^−1^) required to achieve 50% protection of MDBK cells from BVDV induced cytopathogenicity, as determined by the MTT method. ^g^ Compound concentration (µg mL^−1^) required to reduce the viability of mock-infected Vero-76 cells by 50%. as determined by the MTT method. ^h^ Compound concentration (µg mL^−1^) required to reduce the plaque number of hRSVA2, VSV, HSV-1, VV, Sb-1and CV-B4 by 50% in Vero-76 monolayers. * Reference Compds: CC_50_ and EC_50_ are in µM.

## Data Availability

The data used to support the findings of this study are available from the corresponding author upon request.
